# B cells response directed against Cut4 and CFP21 lipolytic enzymes in active and latent tuberculosis infections

**DOI:** 10.1371/journal.pone.0196470

**Published:** 2018-04-30

**Authors:** Wendy Rénier, Arnaud Bourdin, Pierre-Alain Rubbo, Marianne Peries, Luc Dedieu, Sophie Bendriss, Laurent Kremer, Stéphane Canaan, Dominique Terru, Sylvain Godreuil, Nicolas Nagot, Philippe Van de Perre, Edouard Tuaillon

**Affiliations:** 1 Pathogenesis and Control of Chronic Infections, INSERM, EFS, Université de Montpellier, CHU Montpellier, Montpellier, France; 2 PhyMedExp, INSERM, EFS, Université de Montpellier, CHU Montpellier, Montpellier, France; 3 Laboratory of Enzymology at Interfaces and Physiology of Lipolysis, CNRS, Université Aix-Marseille, France; 4 Institute of Research on Infection of Montpellier, CNRS, INSERM, Université de Montpellier, Montpellier, France; Fundació Institut d’Investigació en Ciències de la Salut Germans Trias i Pujol, Universitat Autònoma de Barcelona, SPAIN

## Abstract

**Background:**

Better understanding of the immune response directed against *Mycobacterium tuberculosis* (*Mtb*) is critical for development of vaccine strategies and diagnosis tests. Previous studies suggested that *Mtb* enzymes involved in lipid metabolism, are associated with persistence and/or reactivation of dormant bacilli.

**Methods:**

Circulating antibodies secreting cells (ASCs), memory B cells, and antibodies directed against Cut4 (Rv3452) and CFP21 (Rv1984c) antigens were explored in subjects with either active- or latent-tuberculosis (LTB), and in *Mtb*-uninfected individuals.

**Results:**

Circulating anti-Cut4 ASCs were detected in 11/14 (78.6%) subjects from the active TB group *vs*. 4/17 (23.5%) from the LTB group (p = 0.001). Anti-CFP21 ASCs were found in 11/14 (78.6%) active TB *vs*. in 5/17 (29.4%) LTB cases (p = 0.01). Circulating anti-Cut4 and anti-CFP21 ASCs were not detected in 38 *Mtb* uninfected controls. Memory B cells directed against either Cut4 or CFP21 were identified in 8/11 (72.7%) and in 9/11 (81.8%) subjects with LTB infection, respectively, and in 2/6 *Mtb* uninfected individuals (33.3%). High level of anti-Cut4 and anti-CFP21 IgG were observed in active TB cases.

**Conclusion:**

Circulating IgG SCs directed against Cut4 or CFP21 were mostly detected in patients presenting an active form of the disease, suggesting that TB reactivation triggers an immune response against these two antigens.

## Introduction

With almost 8.6 million cases of tuberculosis (TB) and 1.6 million of deaths related to TB each year, TB remains a major burden worldwide [1]. However, among the estimated third of the world’s population infected by Mycobacterium tuberculosis (Mtb), most of individuals will never develop an active form of the disease in their lifetime. Immunological mechanisms involved in the bacterial control during the latent stage of the infection remain incompletely understood [2]. Better understanding of the immune response directed against TB is critical for development of vaccine strategies and diagnosis tests. Regarding immunodiagnostic of TB, interferon gamma released assays (IGRA) has improved the identification of asymptomatic latent TB (LTB) in low incidence countries [3–4] but were unable to distinguish active from latent TB infection [5–7], and commercial assays based on antibody detection failed to reach the expected performances for a diagnosis assay dedicated to TB diagnosis [8]. It is long known that cellular response is essential to maintain TB in the latent form of the infection, but the B-cell response against *Mtb* antigens has been less studied and there is still a controversy about whether humoral immunity contributes to host defense against TB [2, 9, 10]. The tuberculous granuloma may be viewed as a peripheral lymphoid follicle-like structure displaying features of B cell germinal center [11]. Cellular proliferation is observed in these B cell aggregates in the lungs of humans and mice with TB [12]. Recent evidence suggest that antibody response during TB infections in the latent form is distinct from the humoral response observed in active TB [13, 14].

In previous studies, *Mtb* lipolytic enzymes have been identified as targets of humoral response in active-TB [15, 16]. We have observed antibody directed against CUT4 in subjects with active TB [15]. T cell and B cell response directed against CFP21 has been reported in mice and human [17–20]. In addition, CFP21 has been used in multivalent vaccines and induced protective immunity against Mtb infection in mice [21]. These enzymes are involved in lipid metabolism and could take part in *Mtb* exit of dormancy [22–25]. During the dormancy phase, *Mtb* remains constrained within lung granulomas, which are composed of several cellular types, including T cells, B cells, and macrophages [11, 12, 26, 27]. *Mtb* persists confined toward the center of the granuloma, in foamy macrophages, that are lipid-rich cells derived from normal macrophages [25, 28–30]. The pathogen itself accumulates the host lipids in the form of intra-cytoplasmic lipid inclusions. These lipidic inclusions, mainly composed of triacylglycerides, could be later used as energy substrate by the bacilli during dormancy or in case of reactivation [25, 28–30].

The analysis of B cell immune response directed against TB lipolytic enzymes such as Cut4 and CFP21 offers an opportunity to explore the host-pathogen interplay because these enzymes are immunogenic, secreted by the bacilli [31], and may be expressed during TB reactivation [15, 16]. Cutinases Cut4 and CFP21 are serine-esterases involved in the metabolism of lipidic substrates, together with other lipolytic enzymes [22, 23, 31]. During viral or bacterial infections, antigen exposure triggers the immune response through B cell activation with differentiation either into plasmablasts (CD20^-^, CD38^+++^), and plasma cells (CD20^-^, CD138^+^), or into resting memory B cells. The peaks of circulating plasmablasts and plasma cells can be detected by flow cytometry or by ELISpot techniques as early as seven days after vaccination or infection [32, 33], and remain detectable only as long as the pathogen is actively released from infected cells [34–36]. By contrast, specific memory B cells that play a key role in the long term maintenance of specific immune responses have an estimated half-life exceeding the life expectancy of individuals [37].

This study aimed to explore circulating antibody secreting cells (ASCs), memory B cells, and antibodies directed against two *Mtb* lipolytic enzymes belonging to the cutinase family: Cut4 and CFP21 antigens, in subjects with either active TB disease or LTB infection or no *Mtb* infection.

## Materials and methods

### Study participants

Subjects were enrolled at the Montpellier University Hospital. The population consist of subjects diagnosed with active or latent TB and patients uninfected by *Mtb* but immunized against BCG in childhood. The French BCG vaccination program consisted of a mandatory BCG vaccine dose in children until 2007, and of re-vaccination of tuberculin-negative children. The status for TB was based on the results of *Mtb* cultures, IGRA assays, clinical symptoms presentation and/or chest X-Ray-thoracic CT scan. All the subjects were tested for *Mtb* infection using the T-Spot.®*TB* assay (Oxford Immunotec, UK), an ELISpot assay based on the detection IFN-γ SCs after stimulation with ESAT-6 and CFP10 peptides. Patients were included after approval of the local ethics committee (Sud Méditerrannée III, France) and after providing a written informed consent, (Trial registration: NCT No. 02898623).

### *M*. *tuberculosis* antigens preparation

Cut4 (Rv3452) and CFP21 (Rv1984c) were produced as N-terminal His-tag fusion proteins as previously described [6, 31]. Briefly, genes were amplified from cosmids MTCY13E12 and MTCY39, respectively (Pasteur Institute, Paris, France), and cloned into pDEST™14 expression vector allowing the expression of N-terminal His-tag fusion proteins. All proteins were produced in *E*. *coli* strains, refolded from inclusion bodies and purified using a Ni^2+^-NTA column and gel filtration [31].

For CFP21, the His-tag was removed by enzymatic digestion with TEV and proteins without His-tag were purified by exclusion from a Ni^2+^-NTA column and conserved in Tris 10 mM, NaCl 150 mM, pH 8. Due to its peculiar pH instability and NaCl sensitivity, the Cut4 His-tag was not removed and protein was conserved into 50 mM CHES buffer, pH 9 [31]. Tuberculin Purified Protein Derivative (PPD) was purchased from Statens Serum Institut (Denmark).

### Cells preparation and ELISpot assay

Peripheral Blood Mononuclear Cells (PBMCs) were purified using lymphocytes separation medium (Eurobio, France, following the manufacturer’s indications) and cryopreserved. After thawing, cells were mixed with purified erythrocytes before the addition of the Rosette-Sep cocktail for enrichment of B cells (Stemcell Technologies, France).

Immunobilon-P flat bottomed 96-wells microtiter plates (Millipore, USA) were coated with either phosphate buffered saline (PBS; negative control; Eurobio, France), unlabelled human immunoglobulin G (IgG) (positive control; Rockland Inc, USA), PPD or Cut4 or CFP21 at a final concentration of 50 μg/ml. Plates were then blocked with complete RPMI culture medium (Eurobio) containing 10% FBS (Foetal Bovine Serum, Eurobio) and supplemented with penicillin (100 U/ml) and streptomycin (100 μg/ml) (Invitrogen, UK) for 2 hours at 37°C, in the presence of 5% CO_2_. One hundred thousand B cells/well were seeded, except for the IgG positive control where only five thousand B cells were used. Cells were then incubated with 200 μl of RPMI supplemented by 10% FBS/well for 22–24 hours at 37°C, in the presence of 5% CO_2_. After removal of the cells, 100 μl of secondary antibody directed against human IgG conjugated with alkaline phosphatase (Rockland Inc, USA) was added (1/1000 dilution in PBS, incubated over-night at 4°C). Finally, the immunospots were revealed using NBT-BCIP substrate (BioRad, USA). Enumeration of total IgG secreting cells was used as control of the capacity of the cells to secrete IgG. ASCs were counted using an Axioplan 2 Imaging microscope (Zeiss, Germany) at a magnification of 40×. Results were expressed as the number of immunospots produced by 1×10^5^ B lymphocytes after subtraction of the number of ASCs found in the negative control.

### *In vitro* memory B cells activation

Briefly, purified B cells were activated during a 5-day culture at 37°C, in the presence of 5% CO_2_ using a polyclonal activator cocktail: CpG oligonucleotide ODN 2006 (InVivoGen, CA, USA; 10 μg/ml), IL2 (100 U/ml), IL10 (50 ng/ml), IL21 (0.1 μg/ml) (Peprotech Inc., USA), histidine-tagged-CD40L (0.5 μg/ml), anti-His (R&D Systems, USA; 5 μg/ml), in RPMI with 10% FBS. Enumeration of IgG secreting cells was used as a positive control to evaluate the capacity of B cells to undergo the terminal differentiation into plasma cells under polyclonal stimulation [28]. The positive controls have to display at least 5 spots without polyclonal activation (5,000 B cells) and 7 spots with polyclonal activation (500 B cells). In other wells, 1×10^5^ activated B cells have been needed for the rest of the ELISpot procedures described before.

### Enzyme-Linked ImmunoSorbent assay using *Mtb* lipolytic enzymes as antigens

The 96-wells microtiter plates (Nunc, Denmark) were coated overnight with 100 μl of either tuberculin PPD or Cut4 or CFP21 at a final concentration of 5 μg/ml. Wells were blocked with Dulbecco’s PBS pH 7.4 (Biowest, France), 2% BSA, 0.01% Tween 20 (Sigma-Aldrich, USA). One hundred microliters of patient blood serum per well (dilution: 1/100 in buffer) were incubated at 37°C for one hour. After several washing steps with PBS-T and PBS, bound antibodies were incubated with 100 μl of HRP-conjugated mouse anti-IgG secondary antibody (25 ng/ml) (antibodies-online GmbH, Germany) for an additional 1 hour at 37°C. After washing, 100 μl of enzymatic substrate tetramethylbenzidine (TMB; Bio-Rad, USA) and hydrogen peroxide were added and the reaction was stopped by adding 50 μl of 1 N phosphoric acid. The intensity of the colorimetric signal was measured using a Bio-Rad plate reader at 450 nm.

### Statistical analysis

Data analyses were performed using the SAS version 9.3 software package (SAS Institute, USA). Due to the absence of normal distribution, continuous variables were described as median and inter-quartile range (IQR) and were compared with Wilcoxon Mann-Whitney tests and Chi-square test was used to compare percentages. The Spearman correlation coefficients were calculated to determine the relationship between continuous variables. The results with corresponding *P*-values < 0.05 were considered to be statistically significant.

## Results

### Clinical and biological characteristics of the subjects

The population studied included 49 individuals negative for *Mtb* infection as defined by a negative IGRA, 27 subjects presenting a LTB infection (IGRA positive without evidence of active TB), and 14 subjects with an active TB disease (*Mtb* culture positive). Subjects were untreated for TB at time of sampling. Clinical characteristics are presented in **Table 1**. Among the 27 LTB patients, 5 had a history of active TB and were appropriately treated more than 10 years before sampling and 8 had a clinical suspicion of active TB. Among the 14 patients presenting an active form of the disease, nine were pulmonary cases, four presented both pulmonary and extra-pulmonary localizations and one displayed a strictly gastro-intestinal localization. All active TB cases were confirmed by culture and *Mtb* specific probe utilization. A therapy against active TB was initiated after sampling for all the patients included in the active TB group.

**Table 1:** Subjects characteristics.

	TB negative n = 49	Latent TB n = 27	Active TB n = 14
Age; mean years (± SD)	52.1 (± 20.3)	58.0 (± 20.0)	41.6 (± 22.2)^a^
Female (%)	57.1%	51.9%	21.4%^b^
TB localization; n (%): Pulmonary TB Pulmonary + extra-pulmonary TB Extra-pulmonary TB	Not applicable	Not applicable	9 (64.3%) 4 (28.7%) 1 (7%)
Sputum smear positive (%)	Not applicable	Not applicable	43.0%
T-spot^®^TB; median (IQR) ESAT-6 INF-γ SCs CFP-10 INF-γ SCs	0 (0–0) 0 (0–1)	27 (14–69)^c^ 30 (9–49)^c^	135 (23–200)^d^ 115 (50–180)^e^

IQR: interquartile range.

Chi-Square and Mann-Withney Wilcoxon tests

^a^p = 0.0101 compared to LTB and p = 0.05 compared to TB negative

^b^p = 0.0184 compared to TB negative

^c^p<0.001 compared to TB negative

^d^p<0.001 compared to negative TB and p = 0.0074 compared to LTB

^e^p<0.001 compared to negative TB and p = 0.084 compared to LTB.

### Enumeration of spontaneous circulating ASCs directed against Cut4 and CFP21

**Fig 1:**
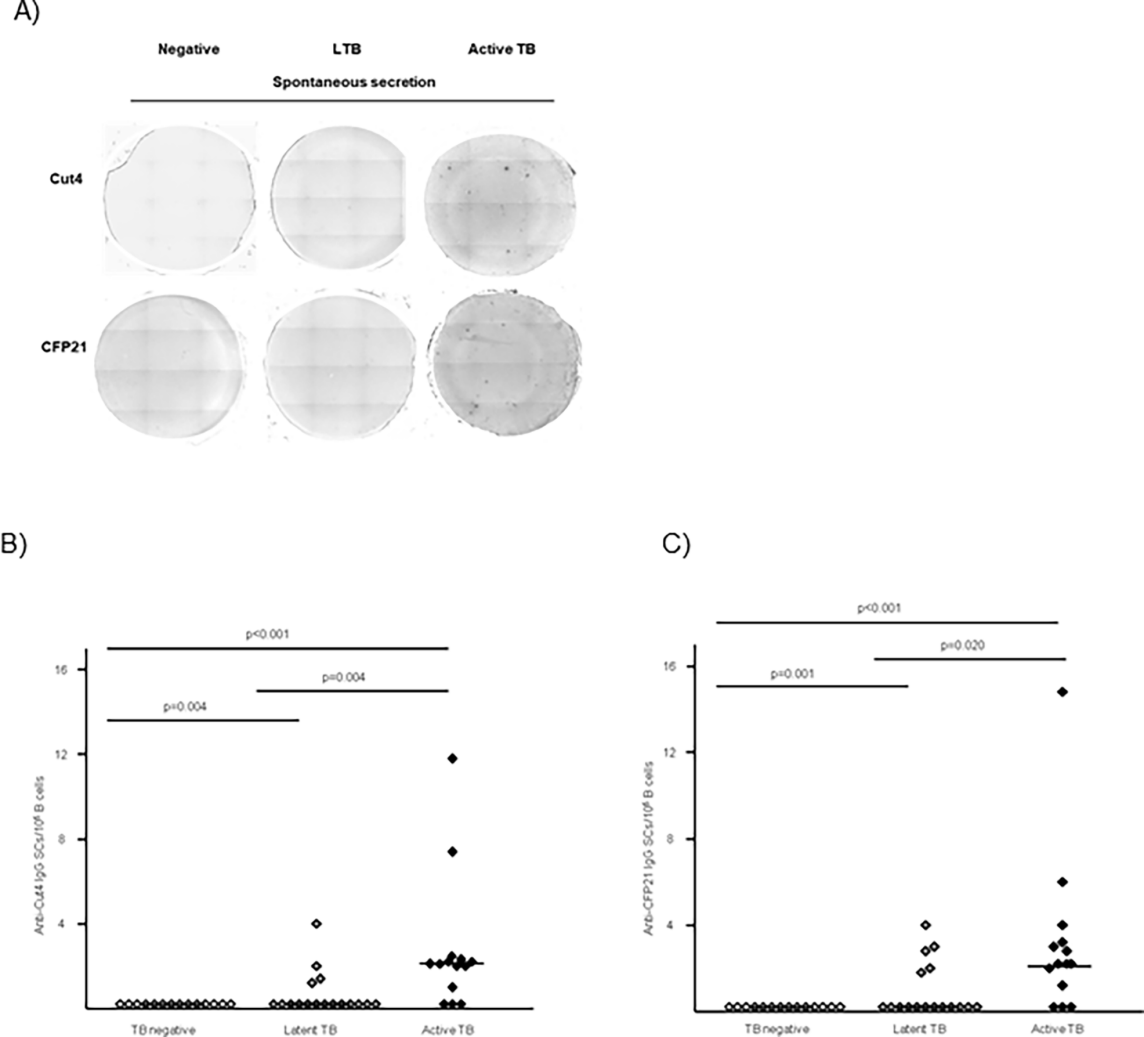
Anti-Cut4 and anti-CFP21 antibody circulating secreting cells (ASCs). (A) Anti-Cut4 and anti-CFP21 ASCs from representative subjects of the three TB groups using ELISpot assay. 0.1 million enriched B cells were seeded per well. Quantification of cells secreting spontaneously anti-Cut4 IgG (B) and anti-CFP21 IgG (C) in Mtb uninfected subjects and subjects with LTB and active TB. The median values have been drawn as horizontal lines. Values of Wilcoxon Mann Withney tests are indicated.

Circulating cells secreting spontaneously IgG antibodies (Abs) directed against Cut4 or CFP21 were explored by ELISpot assays. Immunospots corresponding to the footprint of cells secreting Abs directed against Cut4 or CFP21 were enumerated after overnight culture without polyclonal activation (**Fig 1 and S1 Table**). Anti-Cut4 and anti-CFP21 ASCs were observed in LTB and active TB patients but not in *Mtb*-uninfected controls (**Fig 1*A*, 1*B* and 1*C***). Anti-Cut4 ASCs were detected in 11/14 subjects in the active TB group and in 4/17 patients of the LTB group, respectively (p = 0.004). Anti-CFP21 ASCs were detected in 11/14 patients of the active TB group *vs*. 5/17 subjects of the LTB group (p = 0.020) (**Fig 1*B* and 1*C***). The numbers of anti-Cut4 and anti-CFP21 ASCs displayed a moderate positive correlation (ρ = 0.597; p<0.001, data not shown). No correlation was observed between T-spot.®*TB* results and anti-Cut4 or anti-CFP21 ASCs (ρ = 0.1; p = 0.8, data not shown).

### Memory B cells directed against Cut4, CFP21 and PPD in LTB and *Mtb* uninfected individuals

**Fig 2:**
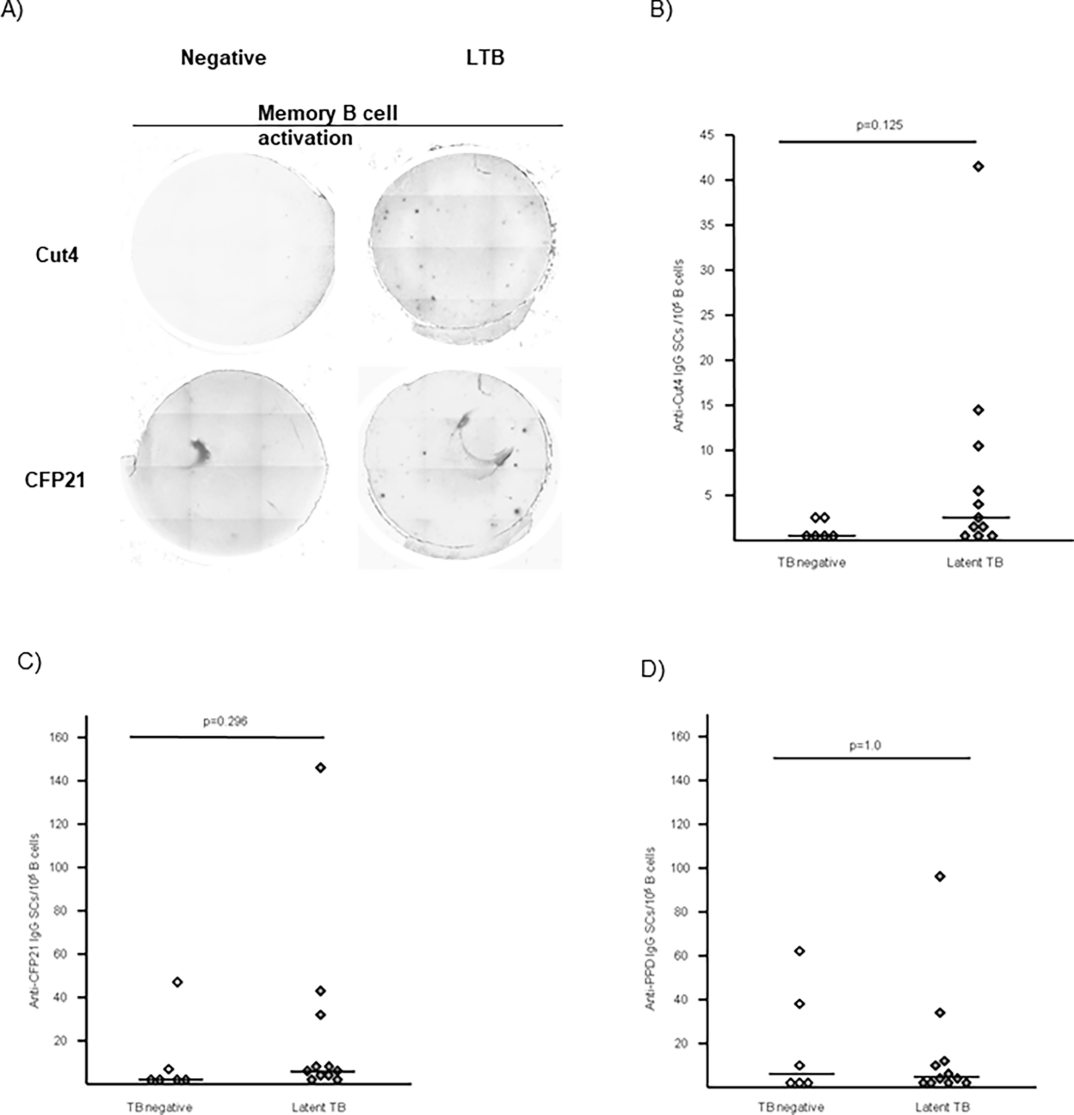
Anti-Cut4 and anti-CFP21 memory B cells. (A) Anti-Cut4 and anti-CFP21 ASCs after polyclonal stimulation from representative subjects of the Mtb uninfected and LTB groups, 1.105 enriched B cells were seeded per well. Quantification of ASC after five days of culture, anti-Cut4 IgG (B) and anti-CFP21 IgG (C) in Mtb uninfected and LTB subjects. The median values have been drawn as horizontal lines. Values of Wilcoxon Mann Withney tests are indicated.

Memory B cells response against Cut4 and CFP21 was explored in 11 LTB patients and six *Mtb* uninfected individuals in whom a second blood collection was performed (**Fig 2*A***). After 5 days of polyclonal activation, the ASCs directed against Cut4 and CFP21 were enumerated. Memory B cells secreting Abs directed against either Cut4 or CFP21 were present in 8/11 and in 9/11 patients with LTB infection, respectively, and in two out of six *Mtb* uninfected individuals (**Fig 2*B* and 2*C***). The number of anti-Cut4 and anti-CFP21 memory B cells detected after polyclonal activation was not correlated (ρ = 0.286, p = 0.266, data not shown).

Memory B cell response against PPD was explored in parallel (**Fig 2*D***). No difference was observed for the number of anti-PPD IgG SCs between the two groups of patients (p = 1). As illustrated by **Fig 2*D***, a number of anti-tuberculin PPD IgG SCs over 10 immunospots per 10^5^ B cells, was enumerated in subjects of TB negative and LTB groups. There was no correlation when comparing Cut4 IgG immunospots with PPD IgG immunospots results (ρ = -0.030; p = 0.909) but a weak positive correlation was found, between CFP21 IgG immunospots and PPD IgG immunospots (ρ = 0.457; p = 0.065; data not shown).

### Serum levels of IgG directed against Cut4, CFP21 and PPD

**Fig 3:**
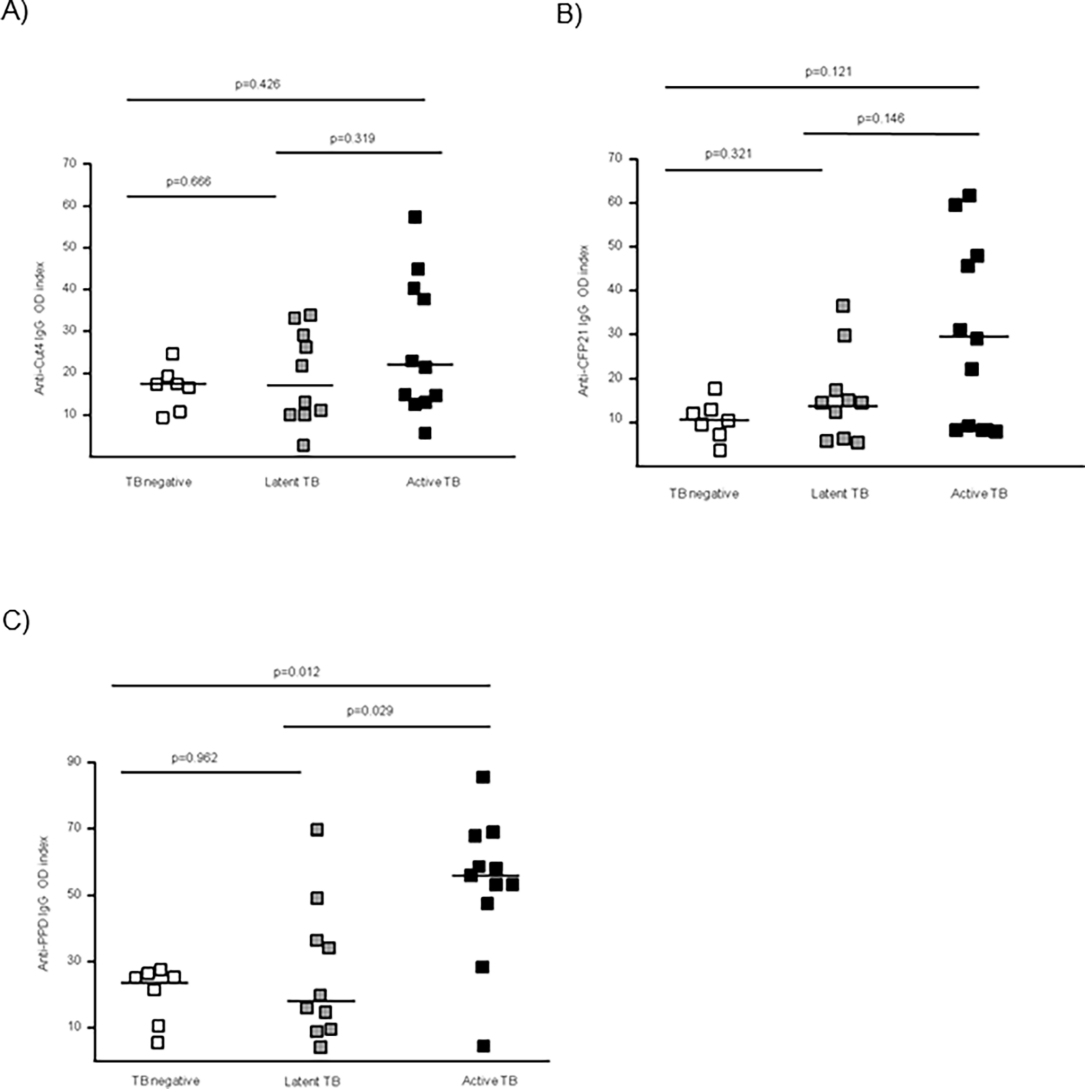
Serum antibody (IgG) levels against CFP21, Cut4 and PPD. The dot plots indicate level of antibodies directed against Cut4 (A), CFP21 (B), and PPD (C) in Mtb uninfected subjects, LTB subjects, and TB subjects. Antibodies levels are indicated per index of the optical density (OD), corresponding to the ratio of sample OD450nm/blank OD450nm.

Levels of IgG anti-Cut4, anti-CFP21 and anti-PPD were explored by ELISA (**Fig 3 and S2 Table**). High index of anti-Cut4 IgG were only observed in the TB group but the mean value was not significantly higher than in the two other groups (p = 0.426, and p = 0.319, respectively), and no difference was observed between LTB and *Mtb* uninfected groups (p = 0.666) (**Fig 3*A***). High level of IgG anti-CFP21 were also observed in some subjects with TB (**Fig 3*B***) and the antibodies levels tend to be higher in subjects with an TB than in LTB and *Mtb* uninfected subjects (p = 0.146 and p = 0.121), respectively. No difference was observed between *Mtb* uninfected and LTB groups (p = 0.321). Higher levels of IgG anti-PPD were observed in the TB group compared to LTB and *Mtb* uninfected groups (p = 0.029, p = 0.012, respectively) (**Fig 3*C***). A strong positive correlation between anti-CFP21 and anti-Cut4 IgG levels was found (ρ = 0.822, p<0.001) whereas the correlations involving tuberculin PPD specific antibodies were only observed for anti-PPD and anti-CFP21 levels (anti-PPD and anti-CFP21 levels: ρ = 0.531, p = 0.004; anti-PPD IgG and anti-Cut4 levels: ρ = 0.433, p = 0.021; data not shown).

## Discussion

Complex and dynamic interplay characterized interaction between bacilli and host immune response during *Mtb* infection. In this study we observed a special pattern of effector B cell response directed against CFP21 and Cut4 *Mtb* antigens and associated with the reactivation phase of TB.

Immunospots, that are the footprints of circulating anti-CFP21 and anti-Cut4 IgG SCs, were found in the majority of subjects with active TB, but only in a limited number of LTB subjects, and were not detected in the control group consisting of *Mtb*-uninfected individuals. Antigen-presentation and terminal differentiation in ASCs can occur in B cells of the granuloma. These cells form aggregates at the surface of the structure and are exposed to TB antigens produced by macrophages [10, 38, 39]. A study in nonhuman primates demonstrates that plasma cells are present in lung granulomas and thoracic lymph nodes and actively secret antibodies specific for Mtb antigens [40]. The generation of circulating plasma cells can be transiently observed in blood following antigen exposure. Hence, the detection of anti-CFP21 and anti-Cut4 IgG SCs can be regarded as an instant snapshot of the immune response to antigen at timing of blood collection. B cell response against others *Mtb* antigens have been previously reported during the different phases of TB. Hence, a higher number of circulating B cells directed against ESAT-6, CFP-10 and Ag85A were observed in patients with active TB compared to LTB and healthy controls [41]. Decay of ASCs directed against lipoarabinomannan and 38kDa antigen have been described during treatment of active TB [42]. Spontaneous release of TB antigen-specific Abs by in vitro-cultured, unstimulated PBMCs have been also reported in children with active TB [43].

In this study, the T-spot.®TB test was used to defined the TB status of the subjects. No correlations between anti-CFP21/Cut4 SCs and IFN-γ SCs directed against ESAT-6/CFP-10 were observed. This assay is based on the enumeration of IFN-γ secreting T cells directed against *Mtb* antigens. ESAT-6 and CFP-10 antigens are expressed in both the latent and active phase of the disease and encoded by the RD1 genetic locus absent from BCG vaccine strains [44]. The peptides used in the T-spot.®TB assay target CD4+ T cells. After short stimulation with ESAT-6 and CFP10 antigens the IFN-γ SCs displayed the CD4+ effector memory cell phenotype (CD45RA-, CCR7-) [45]. We identified subjects with LTB tested positive for ESAT-6 and CFP-10 IFN-γ SCs but negative or positive for anti-CFP21 and anti-Cut4 ASCs. This observation may be a consequence of different Mtb gene expression reflecting metabolic adaptation of the bacilli to different micro-environmental condition at the distinct stages of LTB. During the entrance of the dormancy phase, mycobacteria have been shown to reside within infected foamy macrophages in granulomas, where they can acquire host lipids which accumulate in the form of intracellular lipid-loaded inclusion bodies [30, 46]. The massive accumulation of these lipids may constitute a source of carbon and energy required for bacilli survival during dormancy time-span [25, 26, 30, 47]. The degradation of fatty acids in the bacilli seems to occur during dormancy exit [26, 28]. It has been previously observed that in reactivated bacilli, a reduction in fatty acids levels coincides with an increased lipolytic enzymes activity [26, 48]. Whether Cut4 and CFP21 may be involved in the re-mobilization of the stored intracellular lipids represents an attractive hypothesis that still remains to be demonstrated. About 5–10% of the LTB will undergo a reactivation during their lifetime [48, 49]. A prospective study will need to be performed to establish if stratification of LTB according to anti-CFP2/Cut4 B cell response allows to identified individuals that are at highest risk of developing active TB.

The memory B cells response against Cut4 or CFP21 was also investigated in patients with LTB infection and in individuals uninfected by *Mtb*. Patients with active TB were not tested for memory B cells response because second blood sampling was not possible due to the rapid initiation of the therapy. *In vitro* polyclonal stimulation during a 5-day cell culture step induced the terminal differentiation of memory B cells into ASCs. Memory B cells specific to Cut4 and CFP21 were detected in subjects with a LTB infection whereas their corresponding serum antibodies were not detected. Memory B cells are remarkably long lived cells [37]. The presence of specific memory B cells against Cut4 and CFP21 provides evidence of a previous immune response against these antigens in LTB patients. Surprisingly, a memory B cells response against Cut4 and CFP21 was also observed in two *Mtb* uninfected individuals that were tested negative with the T-spot.®*TB* assay. This observation may be related to a prior *Mtb* infection followed by a spontaneous recovery. Memory B cell response against Cut4 may also result from past immunization with BCG vaccine CFP21 gene located in the RD2 region is deleted in most of BCG vaccine strains [50]. Since Cut4 and CFP21 amino acids sequences share 52% of identity and 66% of homology [25], the two proteins hold common epitopes and cross reaction may have occurred in BCG vaccinated subjects. Circulating memory B cells directed against tuberculin PPD were also detected in both LTB and *Mtb* uninfected subjects. Previous reports have shown that these cells remain detectable several years after BCG vaccine or anti-tuberculous treatment [51–52].

IgG Abs directed against Cut4 and CFP21 were assessed by ELISA. High levels of anti-Cut4 and CFP21 Abs were observed in some of the subjects with TB but the difference between Abs index in the TB group *vs* LTB or *Mtb* uninfected groups did not reach statistical significance. Previous reports have established that serum antibodies directed against CFP21 or Cut4 can be found in patients with active TB whereas low Abs titers were generally observed in LTB infected patients [8]. This discrepancy may be related to the small number of patients analyzed here and to the lower proportion of patients positive for *Mtb* in the sputum compared with the previous study. Indeed, we previously reported that smear positive individuals have highest levels of plasma anti-Cut4 and anti-CFP21 antibodies than smear negative subjects [8].

In conclusion, circulating B cells able to spontaneously generate specific antibodies directed against CFP21 and Cut4 were detected during active TB and in some LTB cases suggesting an ongoing exposure to these antigens. The possible roles of lipolytic enzymes in the physiology of *Mtb* dormancy and it reactivation, as well as their immunogenicity, highlight their possible usefulness as diagnostic and therapeutic targets.

## Supporting information

S1 Table Summary of results obtained with different tests. ‘.’: no test done. ‘+’: positive result for the corresponding test or done for the scan. ‘-‘: negative result for the corresponding test. Status for TB: N: negative; L: latent TB, A: active TB. M: male; F: female. P: pulmonary TB; E-P: extra-pulmonary TB. Samples: f b: fresh blood; fz: frozen PBMCs; fz s: frozen serum.
Without activation: #1 to # 69 included: population analyzed: Negative n = 38, Latent TB n = 17, Active TB n = 14.
With polyclonal B cells activation: # 70 to # 86 included: population analyzed: Negative n = 6, Latent TB n = 11.
Additional negative for ELISA assays: #87 to # 92 included: Negative: n = 6. A Lip B spot assay has been done on a fresh blood sample, PBMCs have been frozen prior to a polyclonal B cells activation and serum or plasma has been collected for ELISA analysis.
Nota bene: ELISA thresholds have been established by analyzing a larger series of sera coming from Montpellier hospital collections (i.e. 286 sera in total). These thresholds correspond to a biological cut-off and correspond to the mean of the negative ratios (OD450nm negative sample / OD450nm blank), plus one standard deviation.
(DOCX)

S2 Table ELISA results obtained with the different antigens. Results are express as index of the optical density (OD), corresponding to the ratio of sample OD450nm/blank OD450nm.
(DOCX)
