# Supplementary material for: B cells response directed against Cut4 and CFP21 lipolytic enzymes in active and latent tuberculosis infections
Source: PLoS One. 2018 Apr 30;13(4):e0196470. doi: 10.1371/journal.pone.0196470 (PMC5927435; doi:10.1371/journal.pone.0196470)
Supplement: S1 Table — ‘.’: no test done. ‘+’: positive result for the corresponding test or done for the scan. ‘-‘: negative result for the corresponding test. Status for TB: N: negative; L: latent TB, A: active TB. M: male; F: female. P: pulmonary TB; E-P: extra-pulmonary TB. Samples: f b: fresh blood; fz: frozen PBMCs; fz s: frozen serum. Without activation: #1 to # 69 included: population analyzed: Negative n = 38, Latent TB n = 17, Active TB n = 14. With polyclonal B cells activation: # 70 to # 86 included: population analyzed: Negative n = 6, Latent TB n = 11. Additional negative for ELISA assays: #87 to # 92 included: Negative: n = 6. A Lip B spot assay has been done on a fresh blood sample, PBMCs have been frozen prior to a polyclonal B cells activation and serum or plasma has been collected for ELISA analysis. Nota bene: ELISA thresholds have been established by analyzing a larger series of sera coming from Montpellier hospital collections (i.e. 286 sera in total). These thresholds correspond to a biological cut-off and correspond to the mean of the negative ratios (OD450nm negative sample / OD450nm blank), plus one standard deviation. (DOCX) [file pone.0196470.s001.docx]

|  | **STATUS** | **Age** | **Gender** | **Culture** | **Sputum smear** | **TB localization** | **Tspot TB** | **IDR** | **Thoracic scan** | **anti-TNFα treatment** | **Cut4 IgG spot** | **CFP21 IgG spot** | **Tuberculin PPD** | **Cut4 ELISA** | **CFP21 ELISA** | **Tuberculin PPD ELISA** | **Type of sample** |
| --- | --- | --- | --- | --- | --- | --- | --- | --- | --- | --- | --- | --- | --- | --- | --- | --- | --- |
| 1 | N | 28 | F | . | . | . | - | . | . | - | - | - | + | . | . | . | f b |
| 2 | N | 52 | M | . | . | . | - | . | . | - | - | - | - | . | . | . | f b |
| 3 | N | 38 | F | . | . | . | - | . | . | - | - | - | - | . | . | . | f b |
| 4 | N | 84 | F | . | . | . | - | . | . | - | - | - | + | . | . | . | f b |
| 5 | N | 59 | F | . | . | . | - | - | . | - | - | - | - | . | . | . | f b |
| 6 | N | 36 | F | . | . | . | - | . | . | - | - | - | + | . | . | . | f b |
| 7 | N | 38 | F | . | . | . | - | . | . | - | - | - | - | . | . | . | f b |
| 8 | N | 67 | M | . | . | . | - | . | . | - | - | - | - | . | . | . | f b |
| 9 | N | 36 | F | . | . | . | - | . | . | - | - | - | - | . | . | . | f b |
| 10 | N | 38 | M | . | . | . | - | - | . | - | - | - | + | . | . | . | f b |
| 11 | N | 49 | M | . | . | . | - | . | + | - | - | - | + | . | . | . | f b |
| 12 | N | 42 | M | . | . | . | - | . | . | - | - | - | + | . | . | . | f b |
| 13 | N | 68 | F | . | . | . | - | . | . | - | - | - | - | . | . | . | f b |
| 14 | N | 49 | F | . | . | . | - | . | . | - | - | - | - | . | . | . | f b |
| 15 | N | 46 | M | . | . | . | - | . | . | - | - | - | + | - | - | - | f b |
| 16 | N | 18 | F | . | . | . | . | . | . | - | - | - | - | . | . | . | f b |
| 17 | N | 37 | F | . | . | . | - | . | . | - | - | - | + | . | . | . | f b |
| 18 | N | 51 | M | . | . | . | - | - | . | - | - | - | + | . | . | . | f b |
| 19 | N | 22 | F | . | . | . | - | - | . | + | - | - | - | . | . | . | f b |
| 20 | N | 69 | F | . | . | . | - | . | + | - | - | - | - | . | . | . | f b |
| 21 | N | 49 | M | . | . | . | - | . | . | - | - | - | + | . | . | . | f b |
| 22 | N | 70 | F | . | . | . | - | . | . | - | - | - | - | . | . | . | f b |
| 23 | N | 48 | M | . | . | . | - | - | . | - | - | - | + | . | . | . | f b |
| 24 | N | 38 | F | . | . | . | - | . | . | - | - | - | + | . | . | . | f b |
| 25 | N | 28 | F | . | . | . | - | . | . | - | - | - | - | . | . | . | f b |
| 26 | N | 29 | F | . | . | . | - | . | . | - | - | - | - | . | . | . | f b |
| 27 | N | 48 | M | . | . | . | - | . | . | - | - | - | + | . | . | . | f b |
| 28 | N | 54 | M | . | . | . | - | . | . | - | - | - | - | . | . | . | f b |
| 29 | N | 85 | M | . | . | . | - | . | . | - | - | - | + | . | . | . | f b |
| 30 | N | 62 | F | . | . | . | - | . | . | - | - | - | - | . | . | . | f b |
| 31 | N | 73 | F | . | . | . | - | . | . | - | - | - | + | . | . | . | f b |
| 32 | N | 91 | M | . | . | . | - | . | . | - | - | - | + | . | . | . | f b |
| 33 | N | 90 | F | . | . | . | - | . | . | - | - | - | + | . | . | . | f b |
| 34 | N | 97 | F | . | . | . | - | . | . | - | - | - | - | . | . | . | f b |
| 35 | N | 32 | M | . | . | . | - | . | . | - | - | - | - | . | . | . | f b |
| 36 | N | 57 | F | . | . | . | - | - | . | - | - | - | + | . | . | . | f b |
| 37 | N | 80 | F | . | . | . | - | . | . | - | - | - | + | . | . | . | f b |
| 38 | N | 55 | M | . | . | . | - | . | . | - | - | - | - | . | . | . | fz |
| 39 | L | 19 | M | . | . | . | . | . | . | - | - | - | + | . | . | . | f b |
| 40 | L | 37 | F | - | - | . | + | + | - | - | - | - | + | . | . | . | f b |
| 41 | L | 69 | F | . | . | . | + | . | . | + | + | - | + | - | - | + | f b |
| 42 | L | 25 | F | . | . | . | + | . | - | - | - | + | - | . | . | . | f b |
| 43 | L | 62 | M | . | . | . | + | . | . | - | + | - | + | - | - | - | f b |
| 44 | L | 28 | F | . | . | . | + | . | . | - | - | - | - | . | . | . | f b |
| 45 | L | 72 | M | . | . | . | + | . | . | - | - | + | - | . | . | . | f b |
| 46 | L | 34 | F | . | . | . | + | . | . | - | - | - | + | . | . | . | f b |
| 47 | L | 70 | F | . | . | . | + | . | . | + | - | - | + | . | . | . | f b |
| 48 | L | 57 | F | . | . | . | + | . | . | - | - | - | - | . | . | . | f b |
| 49 | L | 80 | F | - | - | . | + | . | . | - | - | - | + | . | . | . | f b |
| 50 | L | 89 | F | . | . | . | + | . | . | - | + | + | - | - | - | - | f b |
| 51 | L | 92 | F | . | . | . | + | . | . | - | - | - | - | . | . | . | f b |
| 52 | L | 52 | M | - | - | . | + | . | . | - | + | + | + | . | . | . | f b |
| 53 | L | 82 | M | . | . | . | + | . | . | - | - | - | - | . | . | . | fz |
| 54 | L | 56 | M | . | . | . | + | + | - | + | - | - | - | - | - | - | fz |
| 55 | L | 64 | M | - | - | . | + | . | . | - | - | + | + | . | . | . | fz |
| 56 | A | 66 | M | + | + | P | + | . | + | - | + | + | + | + | + | + | f b |
| 57 | A | 45 | M | + | - | P/E-P | + | + | + | - | + | + | + | - | + | + | fz |
| 58 | A | 25 | M | + | + | P | + | . | + | - | - | + | - | + | + | + | fz |
| 59 | A | 25 | M | + | + | P | + | . | + | - | + | + | + | + | + | + | fz |
| 60 | A | 36 | M | + | + | P | + | . | + | - | + | + | + | - | - | - | fz |
| 61 | A | 22 | M | + | + | P | . | . | . | - | + | - | - | + | + | + | fz |
| 62 | A | 39 | M | + | - | P/E-P | + | . | . | - | + | + | + | - | + | + | fz |
| 63 | A | 33 | M | + | - | P | + | . | + | - | + | + | - | - | - | + | fz |
| 64 | A | 18 | M | + | - | P | + | . | + | - | + | + | + | - | + | - | f b |
| 65 | A | 40 | M | + | - | P/E-P | + | . | - | - | - | + | - | - | - | + | fz |
| 66 | A | 90 | F | + | - | P/E-P | + | . | + | - | + | + | - | . | . | . | fz |
| 67 | A | 49 | F | + | + | P | + | + | + | - | + | - | + | - | - | + | fz |
| 68 | A | 35 | M | + | - | E-P | + | + | - | - | + | + | - | . | . | . | fz |
| 69 | A | 57 | F | + | - | P | + | . | + | - | - | - | + | . | . | . | fz |
| 70 | N | 18 | M | . | . | . | - | . | . | - | - | - | + | . | . | . | fz |
| 71 | N | 54 | M | . | . | . | - | . | . | - | + | - | + | . | . | . | fz |
| 72 | N | 44 | M | . | . | . | - | . | . | - | - | - | - | . | . | . | fz |
| 73 | N | 36 | F | . | . | . | - | . | . | - | - | - | - | . | . | . | fz |
| 74 | N | 23 | M | . | - | . | - | - | . | - | - | + | - | . | . | . | fz |
| 75 | N | 77 | F | . | . | . | - | . | . | + | + | + | - | . | . | . | fz |
| 76 | L | 50 | F | . | . | . | + | + | - | - | + | + | + | . | . | . | fz |
| 77 | L | 27 | M | . | . | . | + | . | . | - | - | + | + | . | . | . | fz |
| 78 | L | 70 | F | . | . | . | + | . | . | + | + | + | + | - | - | - | fz |
| 79 | L | 45 | F | - | - | . | + | . | . | - | + | - | + | . | . | . | fz |
| 80 | L | 62 | F | - | . | . | + | . | . | + | + | + | + | - | - | - | fz |
| 81 | L | 47 | F | - | . | . | + | . | . | - | + | + | + | - | - | - | fz |
| 82 | L | 70 | M | . | . | . | + | + | . | + | - | + | - | + | + | - | fz |
| 83 | L | 82 | M | . | . | . | + | . | . | - | + | + | + | . | . | . | fz |
| 84 | L | 56 | M | - | - | . | + | . | . | - | + | + | + | + | + | - | fz |
| 85 | L | 61 | M | - | . | . | + | . | . | - | + | - | + | + | - | + | fz |
| 86 | L | 83 | M | - | - | . | + | . | . | - | - | + | - | . | . | . | fz |
| 87 | N | 60 | F | . | . | . | - | . | . | + | . | . | . | + | + | - | fz s |
| 88 | N | 42 | M | . | . | . | - | - | . | - | . | . | . | - | - | - | fz s |
| 89 | N | 60 | F | . | . | . | - | . | . | - | . | . | . | - | - | - | fz s |
| 90 | N | 70 | F | . | . | . | - | - | . | - | . | . | . | - | - | - | fz s |
| 91 | N | 44 | M | - | - | - | - | . | . | - | . | . | . | - | - | - | fz s |
| 92 | N | 70 | F | . | . | . | - | . | . | - | . | . | . | - | - | - | fz s |

**S1 Table: Summary of results obtained with different tests**. ‘.’: no test done. ’+’: positive result for the corresponding test or done for the scan. ‘-‘: negative result for the corresponding test. Status for TB: N: negative; L: latent TB, A: active TB. M: male; F: female. P: pulmonary TB; E-P: extra-pulmonary TB. Samples: f b: fresh blood; fz: frozen PBMCs; fz s: frozen serum.

Without activation: #1 to # 69 included: population analyzed: Negative n = 38, Latent TB n = 17, Active TB n = 14.

With polyclonal B cells activation: # 70 to # 86 included: population analyzed: Negative n = 6, Latent TB n = 11.

Additional negative for ELISA assays: #87 to # 92 included: Negative: n = 6.

A Lip B spot assay has been done on a fresh blood sample, PBMCs have been frozen prior to a polyclonal B cells activation and serum or plasma has been collected for ELISA analysis.

*Nota bene*: ELISA thresholds have been established by analyzing a larger series of sera coming from Montpellier hospital collections (*i.e*. 286 sera in total). These thresholds correspond to a biological cut-off and correspond to the mean of the negative ratios (OD_450nm negative sample_ / OD_450nm blank)_, plus one standard deviation.
