# Supplementary material for: B cells response directed against Cut4 and CFP21 lipolytic enzymes in active and latent tuberculosis infections
Source: PLoS One. 2018 Apr 30;13(4):e0196470. doi: 10.1371/journal.pone.0196470 (PMC5927435; doi:10.1371/journal.pone.0196470)
Supplement: S2 Table — Results are express as index of the optical density (OD), corresponding to the ratio of sample OD450nm/blank OD450nm. (DOCX) [file pone.0196470.s002.docx]

|  | Tuberculin | Rv3452 | Rv1984c |
| --- | --- | --- | --- |
| N | 21,504 | 17,389 | 9,525 |
| N | 26,520 | 19,240 | 12,041 |
| N | 10,584 | 10,718 | 3,690 |
| N | 5,676 | 16,762 | 10,464 |
| N | 25,248 | 24,569 | 17,690 |
| N | 26,232 | 9,274 | 7,257 |
| N | 25,204 | 17,333 | 12,845 |
| L | 36,397 | 1,176 | 12,297 |
| L | 34,152 | 29,105 | 36,577 |
| L | 9,656 | 9,986 | 14,432 |
| L | 8,856 | 11,105 | 5,752 |
| L | 16,032 | 13,057 | 5,443 |
| L | 14,736 | 33,884 | 29,752 |
| L | 49,104 | 33,172 | 14,515 |
| L | 69,696 | 26,155 | 17,381 |
| L | 19,896 | 21,742 | 15,113 |
| L | 4,224 | 10,027 | 6,268 |
| A | 49,564 | 5,625 | 8,218 |
| A | 55,962 | 44,864 | 59,577 |
| A | 68,520 | 22,882 | 31,056 |
| A | 58,104 | 21,387 | 29,154 |
| A | 67,762 | 57,333 | 61,584 |
| A | 4,602 | 12,446 | 8,208 |
| A | 28,278 | 14,664 | 22,136 |
| A | 53,295 | 37,685 | 48,235 |
| A | 58,524 | 14,791 | 8,219 |
| A | 53,295 | 40,254 | 45,589 |
| A | 85,584 | 13,037 | 8,948 |

S2 Table: ELISA results obtained with the different antigens. Results are express as index of the optical density (OD), corresponding to the ratio of sample OD450nm/blank OD450nm.
